# *Ab Initio* Speciation of Tc-Gluconate Complexes in Aqueous Systems

**DOI:** 10.1021/acs.inorgchem.4c05115

**Published:** 2025-03-09

**Authors:** Robert Polly, Kathy Dardenne, Sarah Duckworth, Xavier Gaona, Tim Pruessmann, Jörg Rothe, Marcus Altmaier, Horst Geckeis

**Affiliations:** Institut für Nukleare Entsorgung (INE), Karlsruher Institut für Technologie (KIT), Campus Nord, Hermann von Helmholtzplatz 1, 76344 Eggenstein-Leopoldshafen, Germany

## Abstract

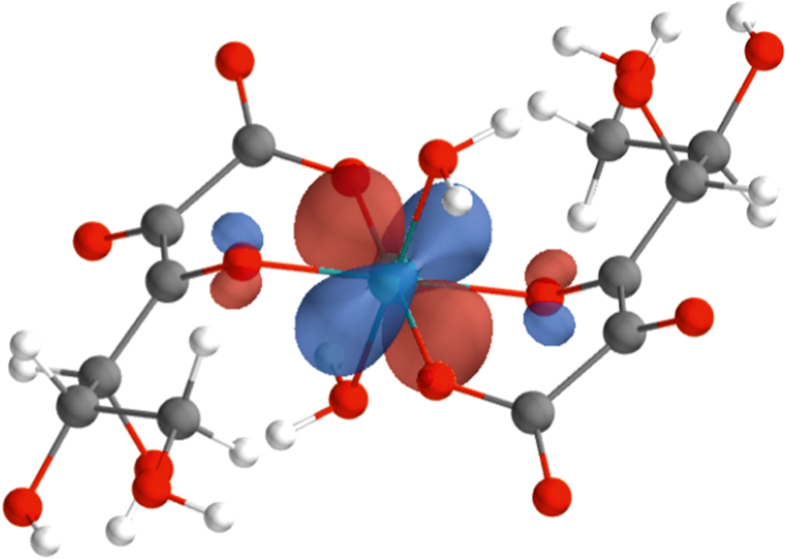

Tc-gluconate complexes in aqueous systems were recently reported and characterized by Tc L_3_-edge X-ray absorption near-edge structure (XANES) measurements [DardenneK.; Inorg. Chem.2021, 60, 12285–1229834328309 10.1021/acs.inorgchem.1c01487]. The puzzling result was reported that the Tc L_3_-edge XANES of the sample containing Tc(IV)-gluconate species differs substantially from that of the Tc(IV)O_2_(am,hyd) hydrous oxide reference sample, whereas the Tc K-edge XANES spectra did not differ significantly. We studied this observation theoretically and tracked the unknown Tc(IV)-gluconate species in a three-step procedure: (1) developing chemical models, (2) optimizing the equilibrium structures of the models, and (3) simulating the corresponding Tc L_3_-edge XANES spectra. We identified the [Tc(IV)(Glu_–2H_)_2_(H_2_O)_2_]^2−^ structure as the most likely Tc(IV)-gluconate species present in our samples and explain the substantial difference between the two Tc L_3_-edge XANES spectra. Additionally, we revisited the Tc(V)-gluconate species and identified the [Tc(V)O(Glu_–H_)_2_]^−^ structure as the most likely Tc(V)-gluconate species in our sample.

## Abstract 2

Accurate quantum chemical calculations allow the speciation of unknown Tc-gluconate in aqueous systems and clarify unexpected changes in the Tc L_3_-edge XANES measurements

## 1 Introduction

Decades of nuclear power generation and nuclear deterrence efforts since the end of World War II have resulted in the accumulation of large amounts of nuclear waste containing a multitude of long-lived radionuclides (i.e., actinides, fission, and activation products). To date, highly active, heat producing nuclear waste forms such as spent nuclear fuel or vitrified reprocessing residues are generally stored temporarily in above-ground facilities. There is international consensus on deep geological disposal of waste forms containing long-lived radiotoxic isotopes. However, details of repository concepts and hypothetical isolation failure scenarios leading to a release of radionuclides into the environment still require extensive research.^1−5^ In this context, a number of long-lived fission products significantly contribute to expected dose rates due to their high yield in the reactor and potential high mobility in the geosphere and biosphere, i.e., ^90^strontium, ^135^cesium, ^129^iodine, ^79^selenium, and ^99^technetium.^6,7^

^99^Tc is a long-lived radioisotope (*t*_1/2_ = 2.121 × 10^5^ a) produced with high yield during nuclear fission in power reactors from the fission of ^235^U and ^239^Pu. A large inventory of this radionuclide is accordingly found in spent nuclear fuel as well as associated with sites for plutonium production or nuclear fuel processing and plays a special role in clean-up efforts, e.g., at the Hanford legacy site.^8−10^ Tc has a very rich redox chemistry with possible oxidation states ranging from −I to +VII^11−15^ where Tc(VII) is the most stable oxidation state under most environmental conditions. In aqueous media, it is predominantly found in the form of a highly mobile TcO_4_^–^ pertechnetate ion. Under more reducing conditions, as expected in deep underground repositories, sparingly soluble Tc hydrous oxides in the oxidation state +IV can be expected. The presence of certain organic ligands (e.g., gluconate, isosacharinic acid, citrate, ethylenediaminetetraacetic acid (EDTA), etc.) may result in the formation of stable aqueous complexes with Tc(IV), eventually increasing the solubility and enhancing the mobility of Tc(IV) (see refs (7) and (16−18) and references therein). Stable complexes and solid phases of Tc(V) with organic ligands are described in the literature^19,20^ as well, although no thermodynamic data is available so far in reference databases.^21,22^

Tc L_3_-edge X-ray absorption near-edge structure (XANES), corresponding to 2p_3/2_ → 4d core excitations, is oxidation-state-sensitive and has been recently shown to identify the oxidation state of Tc in samples containing unknown Tc compounds^7,23,24^ (and references therein). This method is one of the most sensitive methods for accurate Tc oxidation-state and ligand characterization and a leap forward compared to Tc K-edge XANES spectroscopy due to the reduced core-hole lifetime broadening at the shallower L-edges. Moreover, the Tc L_3_ white line position depends directly on the number of 4d electrons that are probed directly by Tc L_3_-edge XANES. Therefore, the Tc L_3_-edge XANES spectra are a very effective tool to characterize the Tc oxidation state.

In our recent work (see Figure 5 in ref (7)), we noticed that Tc L_3_-edge XANES spectra of samples containing Tc(IV)-gluconate complexes in aqueous systems differ substantially from the Tc(IV)O_2_(am,hyd) hydrous oxide reference sample, whereas the Tc K-edge XANES spectra did not differ significantly for both species. The Tc L_3_-edge XANES spectra of Tc(IV)-gluconate feature a double peak structure, whereas the Tc(IV)O_2_(am,hyd) hydrous oxide reference sample displays only one broad peak (see Tc L_3_-edge XANES spectra of sample B and Tc(IV) reference in Figure 5 of ref (7)). Bauters et al.^24^ reported the same observation for [TcCl_4_(PPh_3_)_2_], (NH_4_)_2_[TcBr_6_], (NH_4_)_2_[TcCl_6_], and their Tc(IV)O_2_ reference sample (see Figure 1 in ref (24)). However, these authors did not discuss this observation any further in their work.

Since XANES spectra are frequently used as a fingerprinting tool to identify the oxidation state of the absorbing atom type in a sample, this result is rather puzzling and requires clarification. We attempt this by applying density functional theory (DFT) and Møller–Plesset perturbation theory of second order (MP2) for the optimization of the structures of chemical models of various possible candidates together with relativistic multireference all-electron *ab initio* calculations to simulate the Tc L_3_-edge XANES spectra of the selected chemical models of Tc(IV)-gluconate complexes.

Recently, computational X-ray spectroscopy using either *ab initio* or DFT-based methods has witnessed an enormous advancement^25−52^ and developed into a very reliable tool supporting the understanding of complex X-ray spectra. Alternatively, X-ray spectra of transition metals and actinides are calculated using semiempirical approaches, like the ligand-field multiplet (LFM) semiempirical method,^53−57^ the charge-transfer multiplet (CTM) method,^58,59^ or the crystal-field multiplet theory.^54,55,58,60−62^ Bauters et al.^24^ reported crystal-field multiplet theory simulations of their Tc L_3_-edge XANES spectra of various Tc compounds in the aforementioned work.

This study should help to understand our previous observation and ensure that Tc L_3_-edge XANES spectra of Tc compounds can be used as a reliable tool to identify the oxidation state and further speciation details of unknown Tc species. This can expectedly find relevant applications in the context of nuclear waste disposal, environmental applications (e.g., at legacy sites), and furthermore in fundamental research.

In our initial work,^7^ we left the puzzling discrepancy of the Tc L_3_-edge transition features for Tc(IV)-gluconate and the hydrous Tc(IV)-oxide as an open issue to be solved in the future work.

Note that initial calculations on the Tc(IV)-gluconate system in ref (7) used chemical models based on the optimized [Tc(IV)(Glu_–2H_)_2_(OH)_2_]^4–^ structure but included only the six oxygen atoms interacting with the Tc(IV) ion (the dangling oxygen bonds were saturated with hydrogen atoms as for the Tc(IV)O_2_(am,hyd) system) for the calculations of the Tc L_3_-edge XANES.

The major difference of the present approach compared to our earlier work^7^ is the inclusion of the complete structure of the suggested Tc(IV)-gluconate species in the calculations of the XANES spectra. Moreover, we widened our search for the unknown Tc(IV)-gluconate species by including more possible chemical models in our calculations.

The first step is to develop chemical models for the structures of the Tc(IV)-gluconate complexes from solubility experiments and to optimize these models with DFT and MP2. The averaged Tc–O distances of these optimized structures are compared with the experimental extended X-ray absorption fine structure (EXAFS) results.^7,63^ The structures of the models with an excellent agreement with the experimental Tc–O distances are selected for further theoretical considerations, and in a third step, we calculate the Tc L_3_-edge XANES spectra of these selected models. The calculated XANES spectra are compared with the experimental X-ray spectra.^7^ With this three-step scheme, we attempt to identify the correct Tc(IV)-gluconate structure present in hyperalkaline reducing systems containing gluconate, i.e., sample B in ref (7) ([Tc(VII)]_0_ = 10^–3^ M, 0.1 M NaOH, [Glu^–^] = 0.5 M, and 0.01 M Sn(II)Cl_2_ as the reducing agent).

Although the Tc L_3_-edge XANES of the Tc(V)-gluconate species (sample A in ref (7)) could be simulated using a rather simple [Tc(V)O(OH)_4_]^−^ model,^7^ we revisited this species again in this work and applied the same procedure as described above for the Tc(IV)-gluconate species.

## 2 Methods

### 2.1 Selection of Chemical Models

#### 2.1.1 Tc(IV)-Gluconate Species

In our previous work, we considered only the [Tc(IV)(Glu_–2H_)_2_(OH)_2_]^4–^ species as a chemical model for the unknown Tc(IV)-gluconate species in sample B.^7^ Here, we included more chemical structures in our consideration. Duckworth^16^ and Dardenne et al.^7^ investigated the solubility of TcO_2_(am,hyd) in the presence of gluconate under alkaline to hyperalkaline conditions, both from under- and oversaturation conditions. Solubility experiments were combined with solid-phase characterization by means of X-ray diffraction and X-ray absorption to reveal the fine structure. Two main regions were identified in the solubility data: region I (8 ≤ pH_m_ ≤ 10.5), characterized by a pH-independent solubility behavior, which suggests that no protons are exchanged in the equilibrium reaction that determines solubility and region II (10.5 < pH_m_ ≤ 14), defined by a pH-dependent behavior, possibly involving the release of one H^+^ (see also Figure S1). Based on this evidence, as well as on previous work for the systems Tc(IV)-Glu,^63^ Tc(IV)-ISA,^18^ and Zr(IV)-Glu,^64^ chemical models involving the subsequent formation of the complexes like [Tc(IV)O(Glu_–2H_)]^–^, [Tc(IV)(Glu_–2H_)_2_]^2–^, [Tc(IV)(Glu_–2H_)(Glu_–3H_)]^3–^, and [Tc(IV)(Glu_–2H_)_2_(OH)_2_]^4–^ can be proposed, where Glu_–H_, Glu_–2H_, and Glu_–3H_ correspond to gluconate ligands with one, two, and three deprotonated alcohol groups, respectively. The chemical models with charges *q* = −2, −3 are the most likely candidates, but for completeness, we included models with *q* = −1 and −4 as well. Note that the release of H^+^ in the complexation reaction can result from either deprotonation of the alcohol group or hydrolysis of the water molecules bound to the Tc(IV) core. Both options have been explored in this work. These chemical models have been taken as the basis for the DFT and *ab initio* calculations in this work. It is important to add that the Tc(IV)-gluconate species have *C*_*i*_ inversion symmetry due to the missing pre-edge in the Tc K-edge XANES spectra (see Figure 4 in ref (7)). Hence, we assumed *C*_*i*_ symmetry of all model systems with equal deprotonation of both gluconates in the structure and *C*_1_ symmetry for the other species.

#### 2.1.2 Tc(V)-Gluconate Species

**Figure 1:**
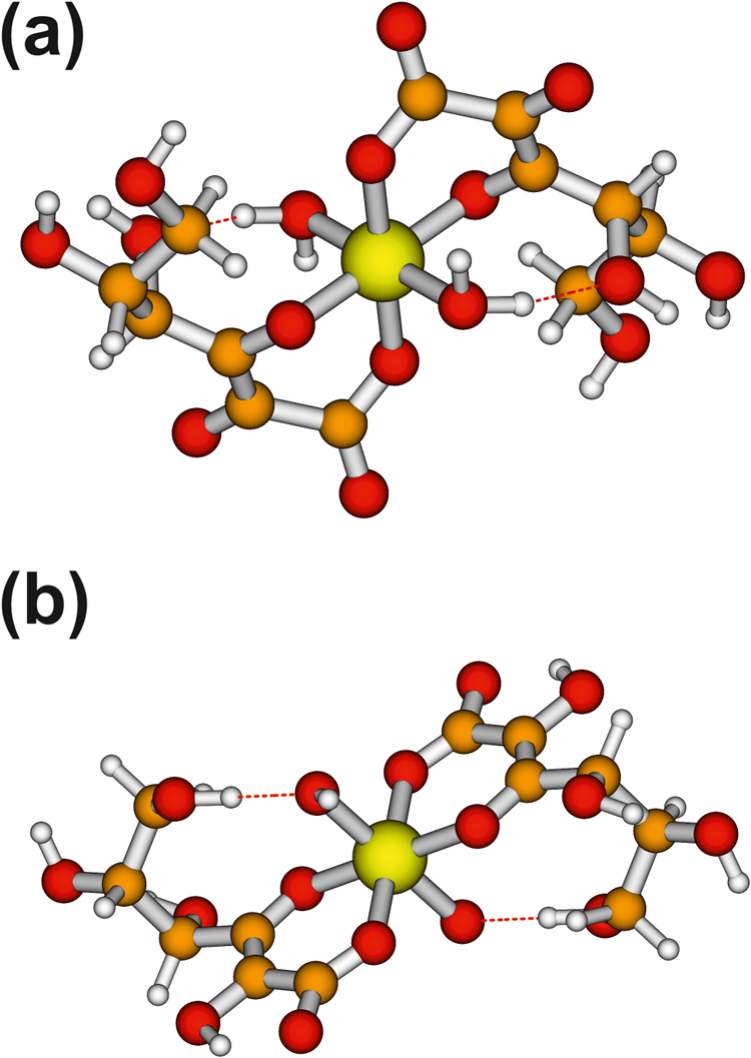
Optimized structures (with MP2/def2-TZVP) of (a) [Tc(IV)(Glu_–2H_)_2_(H_2_O)_2_]^2–^ and (b) [Tc(IV)(Glu_–H_)_2_(OH)_2_]^2–^ for the Tc(IV)-gluconate species.

The initial guess for the chemical models of the Tc(V)-gluconate structure was based on the Tc coordination suggested for (Bu_4_N)[Tc(V)O(O_2_C_6_H_4_)_2_] and Na[Tc(V)O(OCH_2_CH_2_O)_2_] by Davison et al.^19^ (see Figure 1 therein). For the Tc(V)-gluconate species, we selected several possible models with charges varying from +1 to −3, which accounted for different numbers of deprotonated alcohol groups in the gluconate ligands, i.e., [Tc(V)O(Glu_–*n*H_)(Glu_–*m*H_)]^1–(*n*+*m*)^ (*n*, *m* = 0, 1, 2). The K-edge XANES spectra show a pre-edge (see Figure 4 in ref (7)) and hence there is no inversion symmetry in this species. Following the suggested structures,^19^ we assumed *C*_2_ symmetry for the Tc(V)-gluconate species with equal deprotonation of the two gluconates and *C*_1_ symmetry for the other species.

### 2.2 Geometry Optimizations of Chemical Models

The structures of various Tc-gluconate complexes were optimized with TURBOMOLE (www.turbomole.com)^65−72^ on the RI-DFT level using the def2-TZVP^72−74^ basis set followed by optimizations with RI-MP2^75,76^ using the same basis sets. For both methods, we calculated the vibrational frequencies at the equilibrium structure to ensure that there are no imaginary frequencies at the optimized structures.

Since all chemical models are considered in aqueous systems, we performed additional calculations with a conductor-like screening model (COSMO)^77,78^ to check the influence of solvation effects.

### 2.3 Calculations and Simulations of Tc L_3_-Edge XANES Spectra of the Tc-Gluconate Species

Progress in relativistic multireference *ab initio* methods including static and dynamic electron correlation, scalar relativistic effects, and spin–orbit coupling^79−92^ now permits precise electronic structure calculations of electronic states with core electrons excited to nonbonding, antibonding orbitals, or to the continuum. We tackled the calculation of the Tc L_3_-edge XANES spectra of different Tc-gluconate models applying relativistic multireference *ab initio* methods available in MOLCAS8.4.^93^ and OpenMolcas23.06.^94^ The details of the calculation are reported in refs (7,91), and (92) and here we give a brief summary in Section S3.

Experimental spectra were simulated by applying a Lorentzian profile at the calculated transition energies with the intensities given by the oscillator strengths, with a full width at half-maximum γ = 1.6 eV for different models. The value of γ was chosen based on the results reported by Campbell and Papp^95^ for the L_3_-edge of Mo and Ru.

## 3 Results

### 3.1 Selected Chemical Models for the Tc(IV)-Gluconate Species

Based on the solubility experiments combined with X-ray spectroscopy, we selected^7,16^ various chemical models involving Tc(IV)-gluconate complexes with *C*_*i*_ symmetry and with charges varying from *q* = −1 to −4 (see Table 1) to be optimized with DFT/MP2.

**Table 1:** Selected Tc(IV)-Gluconate Models Grouped Together According to Their Total Charge *q*^a^

			agreement with experiment	
charge	label	selected chemical models	averaged Tc–O EXAFS distances^7,63^	Tc L_3_-edge XANES spectra^7^
*q* = −1	1	[Tc(IV)O(Glu_–2H_)]^–^	no	
	2	[Tc(IV)O(Glu_–2H_)]^–^ + 5H_2_O	no	
	3	[Tc(IV)O(OH)(Glu_–H_)]^–^	no	
	4	[Tc(IV)O(OH)_2_(Glu)]^–^	no	
*q* = −2	5	[Tc(IV)(Glu_–2H_)_2_]^2–^	yes	no
	6a	[Tc(IV)(Glu_–2H_)_2_(H_2_O)_2_]^2–^	yes	yes
	6b	[Tc(IV)(Glu_–2H_)_2_(H_2_O)_2_]^2–^ + 4H_2_O	yes	yes
	7a	[Tc(IV)(Glu_–H_)_2_(OH)_2_]^2–^	yes	yes
	7b	[Tc(IV)(Glu_–H_)_2_(OH)_2_]^2–^ + 4H_2_O	yes	yes
*q* = −3	8a	[Tc(IV)(Glu_–H_)(Glu_–2H_)(OH)_2_]^3–^	yes	no
	8b	[Tc(IV)(Glu_–H_)(Glu_–2H_)(OH)_2_]^3–^ + 4H_2_O	yes	no
	9a	[Tc(IV)(Glu_–2H_)(Glu_–3H_)(H_2_O)_2_]^3–^	no	
	9b	[Tc(IV)(Glu_–2H_)(Glu_–3H_)(H_2_O)_2_]^3–^ + 4H_2_O	no	
*q* = −4	10a	[Tc(IV)(Glu_–2H_)_2_(OH)_2_]^4–^	yes	no
	10b	[Tc(IV)(Glu_–2H_)_2_(OH)_2_]^4–^ + 6H_2_O	yes	yes
	11	[Tc(IV)(Glu_–3H_)_2_(H_2_O)_2_]^4–^	no	

a The selected chemical models are shown in column 3. Column 4 indicates whether the averaged calculated Tc–O distances agree with the EXAFS results of Lukens et al.^63^ and Dardenne et al.^7^ Column 5 indicates agreement between the simulated Tc L_3_-edge XANES spectra and those reported by Dardenne et al.^7^ The information in this table provides a summary of the results of the speciation of the Tc(IV)-gluconate species and requires the results presented in Section 3.2 (Table 2 for the equilibrium structures) and Section 3.5.1 (Figure 3 for the simulated Tc L_3_-edge XANES spectra).

Table 1 is central for understanding the results for the speciation of the unknown Tc(IV)-gluconate species. As mentioned above, we employ a three-step procedure: (1) developing chemical models, (2) optimizing the equilibrium structures of the models, and (3) simulating the corresponding Tc L_3_-edge XANES spectra. The first step, the identification of the chemical models, is presented in column 3 entitled “selected chemical models”. The results of the second step, the agreement of the equilibrium structures of the models, are shown in column 4, entitled “averaged Tc–O EXAFS distances” and the results of the third step, the simulation of the Tc L_3_-edge XANES spectra, is presented in column 5, entitled “Tc L_3_-edge XANES spectra”.

### 3.2 Equilibrium Structures of the Selected Chemical Models for the Tc(IV)-Gluconate Species

Lukens et al.^63^ and Dardenne et al.^7^ reported the Tc–O distance of the Tc(IV)-gluconate species from EXAFS measurements to be 201 pm. They are shown together with the theoretical results in Table 2. We used this result as the experimental reference value for the calculated average Tc–O distances.

**Table 2:** Optimized Structures of Tc(IV)-Gluconate Models (See Table 1) 5–7b with *q* = −2, 8a and 8b with *q* = −3, and 10a/b with *q* = −4^a^

experimental results						
						average
sample B (see ref (7))						201^7,63^

theoretical results						
structure	label	method	Tc–O(COO^–^)	Tc–O(CO^–^)	Tc–O(OH_2_/OH^–^)	average
[Tc(IV)(Glu_–2H_)_2_]^2–^	5	DFT	202	206		204
		MP2	200	204		202
[Tc(IV)(Glu_–2H_)_2_(H_2_O)_2_]^2–^	6a	DFT	202	207	217	209
		MP2	207	198	212	206
[Tc(IV)(Glu_–2H_)_2_(H_2_O)_2_]^2–^ + 4H_2_O	6b	DFT	201	205	216	207
		MP2	205	199	211	205
[Tc(IV)(Glu_–H_)_2_(OH)_2_]^2–^	7a	DFT	205	204	206	205
		MP2	207	205	202	205
[Tc(IV)(Glu_–H_)_2_(OH)_2_]^2–^ + 4H_2_O	7b	DFT	204	203	207	205
		MP2	207	205	202	205
[Tc(IV)(Glu_–H_)(Glu_–2H_)(OH)_2_]^3–^+4H_2_O	8a	DFT	203/211	197/205	201/207	204
[Tc(IV)(Glu_–H_)(Glu_–2H_)(OH)_2_]^3–^	8b	DFT	203/206	195/206	206/207	204
[Tc(IV)(Glu_–2H_)_2_(OH)_2_]^4–^	10a	DFT	207^7^	207^7^	199^7^	204^7^
		MP2	208	207	199	205
[Tc(IV)(Glu_–2H_)_2_(OH)_2_]^4–^ + 6H_2_O	10b	DFT	203	204	203	204
		MP2	199	199	198	199

a Various Tc–O distances are listed here: Tc–O(COO^–^) denotes the distance between Tc and the closest oxygen in the deprotonated acid group, Tc–O(CO^–^) to the oxygen in the deprotonated alcohol group, and Tc–O(OH_2_/OH^–^) to an oxygen either in a water molecule or a hydroxide anion. With this detailed information, we calculated the averaged Tc–O distance to be compared with the experimental EXAFS measurements. The results of DFT(BP86) and MP2 calculations are shown (bond lengths are given in pm).

Tc(IV)-gluconate species have to have *C*_*i*_ inversion symmetry due to the missing pre-edge in the K-edge XANES spectra (see Figure 4 in ref (7)). Due to the inversion symmetry, models should be selected with the two gluconate ligands having the same number of deprotonated alcohol groups. Nevertheless, we also included models (e.g., [Tc(IV)(Glu_–H_)(Glu_–2H_)(OH)_2_]^3–^) with different deprotonation of the two gluconate ligands in our consideration and optimized these structures in *C*_1_ symmetry, since a slight deviation from *C*_*i*_ symmetry cannot be excluded. The 10 models displayed in Table 1 labeled 6a/b, 7a/b, 8a/b, 9a/b, and 10a/b differ only by the addition of water molecules interacting with gluconate and are therefore very similar to each other.

We present the results grouped according to the charges of different chemical models for the unknown Tc-gluconate species in sample B.^7^ The criterion for a model to be selected was a maximum deviation from the experimental result of ±5 pm for the average distance of the MP2 structures (or ±10 pm for the DFT structures).

All of the structures of the selected models with charge *q* = −1 have averaged Tc–O distances that differ significantly from the experimental result^7,63^ (see Table S1). Moreover, all models show such a large splitting between the [Tc=O] and the other Tc–O distances that it should be resolved by the EXAFS measurements based on the available *k* (photoelectron momentum) space as two distinct distances. Therefore, all of the models with charge *q* = −1 labeled with 1–4 in Table 1 are excluded from the XANES spectra calculations.

The results for the optimization for the selected models with charge *q* = −2 are summarized in Table 2 and the optimized structures for [Tc(IV)(Glu_–H_)_2_(OH)_2_]^2–^ and [Tc(IV)(Glu_–2H_)_2_(H_2_O)_2_]^2–^ (with MP2/def2-TZVP) are shown in Figure 1. We report the distances from Tc to the oxygens of different functional groups of the gluconates in Table 2 together with the average value of all of the calculated Tc–O distances. It is the latter that has to be compared with the experimental EXAFS results.^7,63^ In general, DFT bond distances are slightly too long. The mean error of the calculated averaged DFT Tc–O distances for the models listed in Table 2 is ≈2.5% with a maximum deviation of ≈4%. This is in excellent agreement with the experimental results. The MP2 results are slightly better, with an average error of ≈2.1%. It is remarkable that the individual Tc–O distances obtained with DFT and MP2 agree very well with only one exception. The Tc–O(H_2_) distances shrink by 5 pm from DFT to MP2. This is related to the correct description of the dispersion interaction with MP2, which is lacking in DFT. As a summary, we observe good agreement of all models labeled as 5–7b with *q* = −2 with the experimental data, as indicated in column 4 of Table 1.

For the two structures [Tc(IV)(Glu_–H_)_2_(OH)_2_]^2–^ and [Tc(IV)(Glu_–2H_)_2_(H_2_O)_2_]^2–^ shown in Figure 1, we performed additional MP2 calculations including COSMO to study the effect of solvation. The deviations from the results presented in Table 2 are 1 pm at most and, therefore, are negligible.

The results of the optimization for the selected models with charges *q* = −3 and −4 are collected in Table 2 as well. Optimizing the two [Tc(IV)(Glu_–2H_)(Glu_–3H_)(H_2_O)_2_]^3–^(+4H_2_O) models (labeled 9a/b) always resulted in [Tc(IV)(Glu_–H_)(Glu_–2H_)(OH)_2_]^3–^(+4H_2_O) (labeled 8a/b) final structures. Therefore, the models labeled 9a/b are discarded and are not included in Table 2. As for the models with charge *q* = −2, the average Tc–O distances of the [Tc(IV)(Glu_–H_)(Glu_–2H_)(OH)_2_]^3–^(+4H_2_O) (labeled 8a/b) models for DFT and MP2 agree very well with the experimental result and are selected for further calculations (see column 4 of Table 1). For *q* = −4, the [Tc(IV)(Glu_–3H_)_2_(H_2_O)_2_]^4–^ model (labeled 11) is not included since the optimizations of the structures of these models always resulted in a [Tc(IV)(Glu_–2H_)_2_(OH)_2_]^4–^ model (labeled 10a). The optimized structures of the [Tc(IV)(Glu_–2H_)_2_(OH)_2_]^4–^(+4H_2_O) models are close to the experimental data, and we selected both models 10a/b for further simulations of the Tc L_3_-edge XANES spectra. In general, DFT bond distances are slightly too long, but all of the optimized structures have average Tc–O distances with excellent agreement with the experimental results. The same holds for the MP2 results, which all give the same or a slightly shorter bond length. For both charges *q* = −3 and −4, the accuracy is as good as for the charge *q* = −2 and reproduce the EXAFS value of the Tc–O distance with great accuracy.

As can be seen from Table 1 (column 4) with the optimization of different models, we narrowed the possible structures for the unknown Tc(IV)-gluconate from 16 down to 9 candidates. These nine models are used for further theoretical considerations.

### 3.3 Selected Chemical Models for the Tc(V)-Gluconate Species

Inspired by the suggested structures of Davison et al.,^19^ we investigated various Tc(V)-gluconate chemical models: [Tc(V)O(Glu_–*n*H_)(Glu_–*m*H_)]^+1–(*n*+*m*)^ (*n*, *m* = 0, 1, 2) (see Table 3). In these chemical models, we have one oxygen directly bound to Tc and two gluconate ligands interacting with this [Tc=O]^3+^ unit.

**Table 3:** Selected Tc(V)-Gluconate Models [Tc(V)O(Glu_–*n*H_)(Glu_–*m*H_)]^+1–(*n*+*m*)^ (*n*, *m* = 0, 1, 2) with Charges *q* = +1,..., −3^a^

			agreement with experiment	
charge	label	selected chemical models	Tc–O EXAFS distances^7,63^	Tc L_3_-edge XANES spectra^7^
*q* = +1	1	[Tc(V)O(Glu)_2_]^+^	no	
*q* = 0	2	[Tc(V)O(Glu_–H_)(Glu)]^0^	no	
*q* = −1	3	[Tc(V)O(Glu_–H_)_2_]^–^	yes	yes
*q* = −2	4	[Tc(V)O(Glu_–2H_)(Glu_–H_)]^−2^	yes	no
*q* = −3	5	[Tc(V)O(Glu_–2H_)_2_]^−3^	yes	no

a The selected chemical models are shown in column 3. Column 4 indicates whether the two different calculated Tc–O distances agree with the two different EXAFS Tc–O distances as determined by Dardenne et al.^7^ Column 5 indicates agreement between the simulated Tc L_3_-edge XANES spectra and those reported by Dardenne et al.^7^ The information in this table provides a summary of the results of the speciation of the Tc(V)-gluconate species and requires the results presented in Section 3.4 (Table 4 for the equilibrium structures) and Section 3.5.2 (Figure 4 for the simulated Tc L_3_-edge XANES spectra).

Table 3 has the same role for the unknown Tc(V)-gluconate species as Table 1 (see Section 3.1) for the unknown Tc(IV)-gluconate species.

### 3.4 Equilibrium Structures of the Selected Chemical Models for the Tc(V)-Gluconate Species

For the Tc(IV)-gluconate species, we showed that the DFT and MP2 results are very close to each other. Therefore, we optimized the structures of the Tc(V)-gluconate species only with DFT.

Dardenne et al.^7^ reported for the Tc(V)-gluconate two clearly distinct Tc–O distances from EXAFS measurements. The [Tc=O] is 164 pm, and the averaged Tc–O distance to the gluconate is 197 pm. They are shown together with the theoretical results in Table 4 and we use these results as the experimental reference values for the calculated Tc–O distances for the possible Tc(V)-gluconate structures.

**Table 4:** Optimized Structures of Tc(V)-Gluconate [Tc(V)O(Glu_–*n*H_)(Glu_–*m*H_)]^+1–(*n*+*m*)^ (*n*, *m* = 0, 1, 2) Models 1–5 (See Table 3)^a^

experimental results					
		[Tc=O]			average Tc–O_1,2_
sample A (see ref (7))		164			197^7^

theoretical results					
structure	label	[Tc=O]	Tc–O_1_(COO^–^)	Tc–O_2_(CO^–^)	average
[Tc(V)O(Glu)_2_]^+1^	1	171	189	206	197
[Tc(V)O(Glu)(Glu_–H_)]^0^	2	167	193	194	193
[Tc(V)O(Glu_–H_)_2_]^1–^	3	169	195	204	199
[Tc(V)O(Glu_–H_)(Glu_–2H_)]^−2^	4	170	199	209	199
[Tc(V)O(Glu_–2H_)_2_]^−3^	5	174	194	202	198

a Various Tc–O distances are listed here, and Tc–O(COO^–^) denotes the distance between Tc and the closest oxygen in the deprotonated acid group and Tc–O(CO^–^) to the oxygen in the deprotonated alcohol group. [Tc=O] is the bond distance to the oxygen directly bound to Tc(V). With this detailed information, we calculated the averaged Tc–O distance to be compared with the experimental EXAFS measurements. The results of the DFT(BP86) calculations are shown (bond lengths in pm).

The results of the optimization for the selected model with charge *q* = +1 are shown in Table 4. We find that the two Tc–O distances are split by 17 pm (see Table 4, Tc–O(COO^–^) = 189 pm and Tc–O(CO^–^) = 206 pm). As before, for the Tc(IV)-gluconate species with *q* = −1, this splitting should be resolved by the EXAFS measurements as two distinct distances. Therefore, we can discard the [Tc(V)O(Glu)_2_]^+^ model system for the Tc(V)-gluconate species. For the optimized structure with charge *q* = 0, we found only 3 Tc–O bonds and the average Tc–O distance of 193 pm is significantly smaller than the experimental value (usually DFT predicts bond lengths that are too long). Therefore, we discarded this chemical model as well.

**Figure 2:**
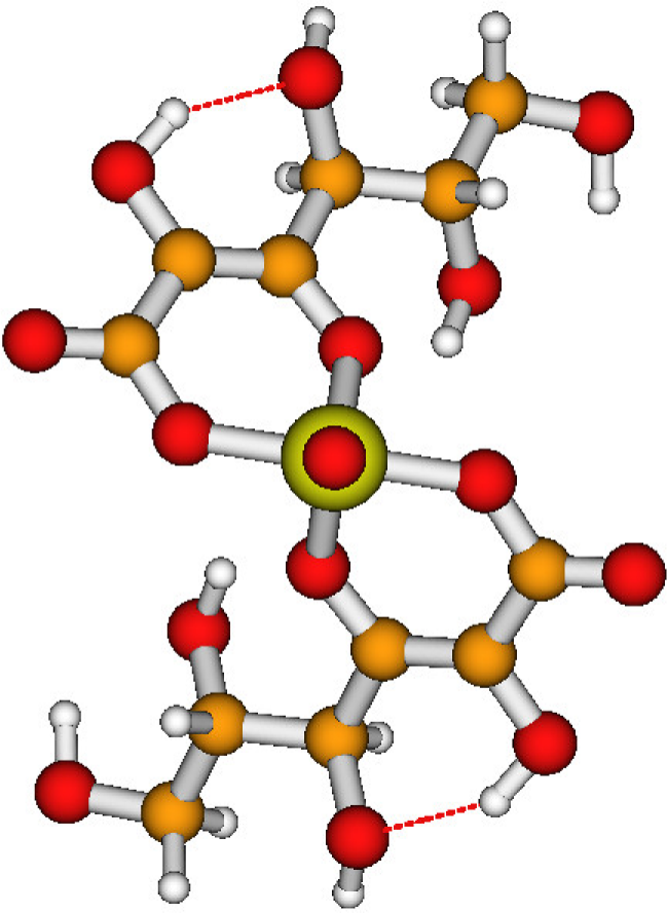
Top view of the optimized structures (with DFT/def2-TZVP) of the [Tc(V)O(Glu_–H_)_2_]^1–^ model along the [Tc=O] bond.

The results of the optimization for the selected models with charges *q* ≤ −1 are summarized in Table 4 as well. For all three charges, we obtain a [Tc=O] distance in very good agreement with the experimental data^7^ with the largest error for the Tc(V)-gluconate system with *q* = −3. The averaged Tc–O distances excellently agree with the experimental data, and the splittings between the Tc–O values are so small that they cannot be resolved anymore by the EXAFS measurements. The optimized structures of all three species agree very well with the experiment, and they are all considered for further investigations; we calculated the Tc L_3_-edge XANES for these three species. Figure 2 shows the optimized structure of the [Tc(V)O(Glu_–H_)_2_]^1–^ species. With this, we reduced the number of possible candidates for the Tc(V)-gluconate species to three structures with *q* ≤ −1 (see Table 3, column 4).

### 3.5 Calculation of the Tc L_3_-Edge XANES Spectra of the Selected Tc-Gluconate Models

#### 3.5.1 Tc(IV)-Gluconate Models

We calculated the Tc L_3_-edge XANES spectra of all of the nine Tc(IV)-gluconate models listed in Table 1 and marked with yes in column 4, which are possible candidates for the observed Tc(IV)-gluconate species in sample B.^7^ The list of included states in the calculations for all of the species with *C*_*i*_ symmetry is shown in Table S2. The calculations consider the lowest lying doublet and quartet states in A_g_ symmetry with a full 2p core–shell together with 450 core-excited spin-free states (see Section S4) in A_u_ symmetry. Upon inclusion of the spin–orbit coupling, we have 840/420 spin–orbit-coupled core-excited states of the Tc L_3_/L_2_-edge manifold included in the calculations. We determined the excitation energies and oscillator strengths, which are used in turn to simulate the Tc L_3_-edge XANES spectra.

The simulated Tc L_3_-edge XANES spectra of the [Tc(IV)(Glu_–2H_)_2_]^2–^ model (labeled 5 in Table 1) show the same shape as the Tc(IV)O_2_(am,hyd) with only one intense peak (see Figure 5 in ref (7)) and can therefore be excluded as a possible candidate for the Tc(IV)-gluconate species in sample B.^7^

**Figure 3:**
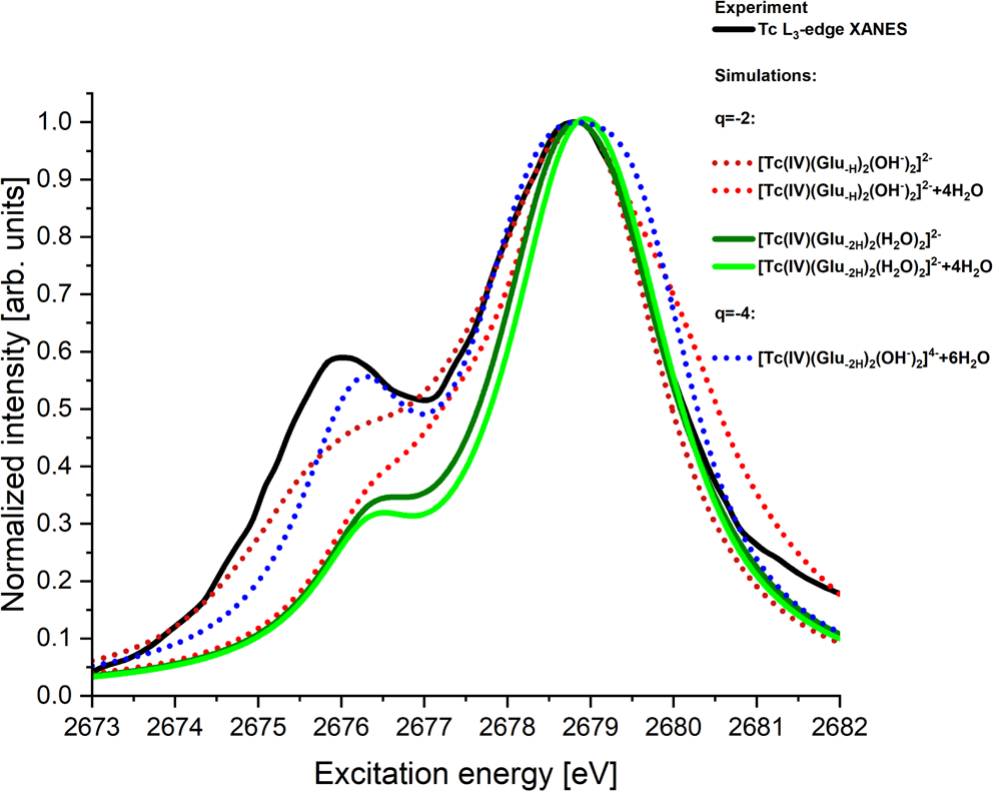
Simulated Tc L_3_-edge XANES spectra of selected Tc(IV)-gluconate models are listed in Table 1 (marked with yes in column 5). All simulated spectra have been individually shifted by ≈20 eV so that they align with the maximum of the experimental result.

In contrast to that, the simulated Tc L_3_-edge XANES spectra of all of the other four models (labeled 6a/b and 7a/b in Table 1) show a shape similar to the experimental spectra with two peaks (see Figure 3). Hence, for the charge *q* = −2, four candidates remain for our Tc(IV)-gluconate species. But the simulated Tc L_3_-edge XANES spectra for [Tc(IV)(Glu_–2H_)_2_(H_2_O)_2_]^2–^(+4H_2_O) (labeled 6a/b) agree clearly better (see Figure 3 solid green lines) with the experimental data compared to [Tc(IV)(Glu_–H_)_2_(OH)_2_]^2–^(+4H_2_O) (labeled 7a/b; see Figure 3, dotted red lines). An additional information can be gained from the simulations pointing toward the [Tc(IV)(Glu_–2H_)_2_(H_2_O)_2_]^2–^(+4H_2_O) species. The first shoulder in [Tc(IV)(Glu_–H_)_2_(OH)_2_]^2–^ disappears when explicit water molecules (+4H_2_O) are taken into account in the simulations. This is not observed for [Tc(IV)(Glu_–2H_)_2_(H_2_O)_2_]^2–^(+4H_2_O) species. This is another argument why the [Tc(IV)(Glu_–2H_)_2_(H_2_O)_2_]^2–^(+4H_2_O) (labeled 6a/b) structure is the more likely candidate for the unknown Tc(IV)-gluconate species, but [Tc(IV)(Glu_–H_)_2_(OH)_2_]^2–^ cannot be excluded.

For the charge *q* = −3, we simulated the Tc L_3_-edge XANES spectra of the [Tc(IV)(Glu_–H_)(Glu_–2H_)(OH)_2_]^3–^ model (labeled 8a/b). But the simulated XANES spectra did not reproduce the observed Tc L_3_-edge XANES spectra at all, and therefore this model can be removed from the list of possible candidates in our solution.

For the charge *q* = −4, we obtained two very different spectra for the two considered models. The simulated Tc L_3_-edge XANES spectra of the [Tc(IV)(Glu_–2H_)_2_(OH)_2_]^4–^ model (labeled 10a in Table 1) show the same shape as the Tc(IV)O_2_(am,hyd) with only one intense peak. In contrast to that, the simulated Tc L_3_-edge XANES spectra of the [Tc(IV)(Glu_–2H_)_2_(OH)_2_]^4–^ + 6H_2_O model (labeled 10b in Table 1; see Figure 3, dotted blue lines) show a shape similar to the experimental measurement with two peaks (see Figure 3). Hence, for charges *q* = −3 and −4, only the [Tc(IV)(Glu_–2H_)_2_(OH)_2_]^4–^ + 6H_2_O model remains as a possible candidate for our Tc(IV)-gluconate species. However, the simulated XANES spectra of this model strictly require the presence of six water molecules since the simulated spectrum of the [Tc(IV)(Glu_–2H_)_2_(OH)_2_]^4–^ model does not reproduce the measured Tc L_3_-edge XANES spectra of the Tc(IV)-gluconate species at all. Therefore, it is less likely that [Tc(IV)(Glu_–2H_)_2_(OH)_2_]^4–^ + 6H_2_O is a candidate for the Tc(IV)-gluconate species present in our measurements.

Another guide to decide which model is best suited for the unknown Tc(IV)-gluconate species is peak splitting between the main peak and second peak with a lower intensity. The experimental value is 2.9 eV and the calculated values of the splitting vary from 2.2 to 2.5 eV for the three models under consideration. Hence, this information does not provide further evidence to track the unknown Tc(IV)-gluconate species.

As mentioned in Section 2.1.1, the chemical models with charges *q* = −2 and −3 are the most likely candidates based on solubility experiments, but for completeness, we included models with *q* = −1 and −4 as well. Hence, we can exclude the candidate with *q* = −4 based on further experimental evidence.^7,16^ The predominance of this complex in the aqueous phase would define a slope of +2 for the solubility equilibrium described in reaction 1
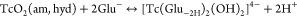
1

This is in contrast to the experimental observations^7,16^ where a slope of 0 and +1 in the alkaline to hyperalkaline conditions was reported (see Figure S1).

As a conclusion, we identify the [Tc(IV)(Glu_–2H_)_2_(H_2_O)_2_]^2–^ model as most likely to be the candidate for the Tc(IV)-gluconate species as found by Dardenne et al.^7^

#### 3.5.2 Tc(V)-Gluconate Models

We calculated the Tc L_3_-edge XANES spectra of all three Tc(V)-gluconate models listed in Table 4 and marked with yes in column 4.

**Figure 4:**
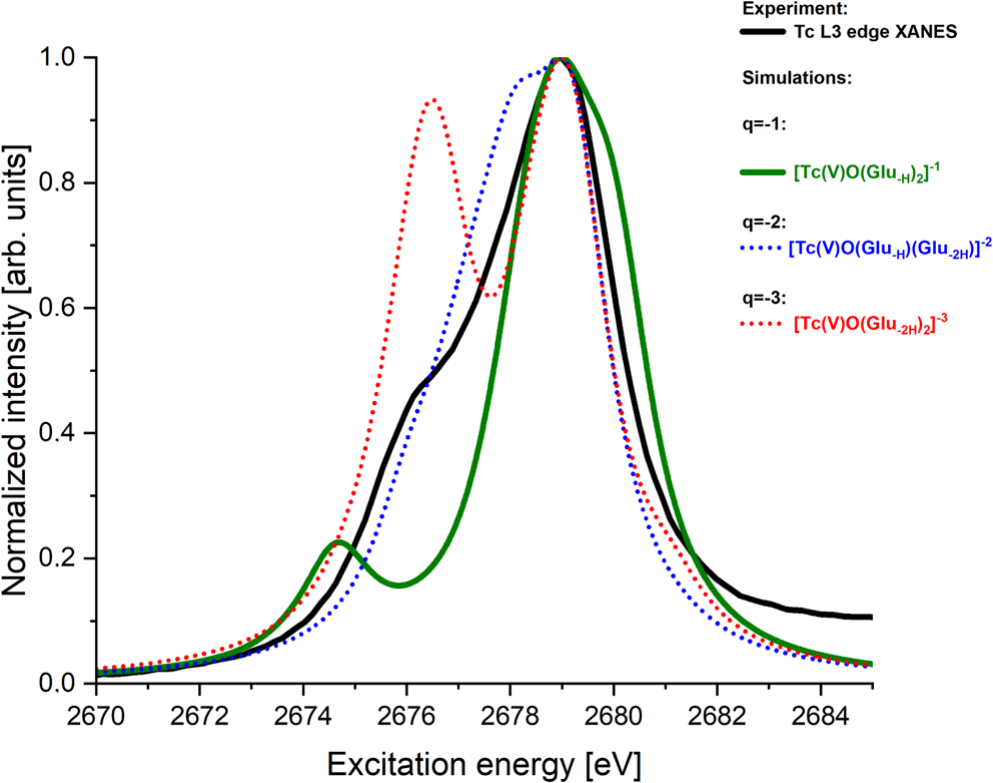
Simulated Tc L_3_-edge XANES spectra of selected Tc(V)-gluconate models are listed in Table 4 (marked with yes in column 5). All simulated spectra have been individually shifted by ≈20 eV so that they align with the maximum of the experimental result.

The simulated Tc L_3_-edge XANES spectra of all of the models (labeled 3–5 in Table 4) are shown in Figure 4. Hence, we single out a candidate for the Tc(V)-gluconate species: [Tc(V)O(Glu_–H_)_2_]^1–^. The simulated Tc L_3_-edge XANES spectrum is most similar to the experimental XANES, and therefore we identify it as the most likely species present in sample A.^7^ The ground state of this species is a triplet state (4d^2^) as expected and needs no further consideration.

### 3.6 Characterization of the Ground and Relevant Core-Excited States of the [Tc(IV)(Glu_–2H_)_2_(H_2_O)_2_]^2–^(+4H_2_O) Model

#### 3.6.1 Ground State

The fundamental difference of the Tc L_3_-edge XANES spectra of the Tc(IV)O_2_(am,hyd) hydrous oxide reference sample to sample B^7^ with the Tc(IV)-gluconate species can be reproduced with the *ab initio* calculations of the XANES spectra. The explanation for this change is provided by the calculations as well. For this, we looked at the electronic structure of the ground state of both species.

As shown by Dardenne et al.,^7^ the electronic structure of Tc in the ground state of Tc(IV)O_2_(am,hyd) has 4d^3^ occupation with *S* = 3/2. All of the three spins of the electrons occupying the 4d orbitals in T_2g_ (see Figure 8c in ref (7)) are parallel, which can be easily explained by the ligand-field splitting of the 4d orbitals in an octahedral environment.

**Figure 5:**
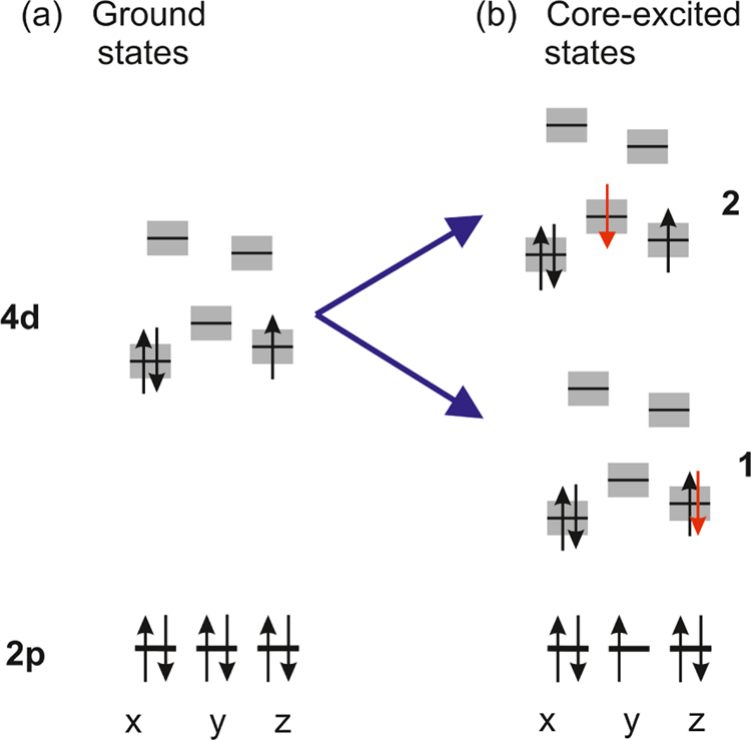
Schematic MO diagram of the doublet ground and doublet core-excited states of the [Tc(IV)(Glu_–2H_)_2_(H_2_O)_2_]^2–^ model. The lowest doubly occupied 4d_π_ orbital is shown in Figure 6a and the singly occupied 4d_δ_ orbital is shown in Figure 6b.

**Figure 6:**
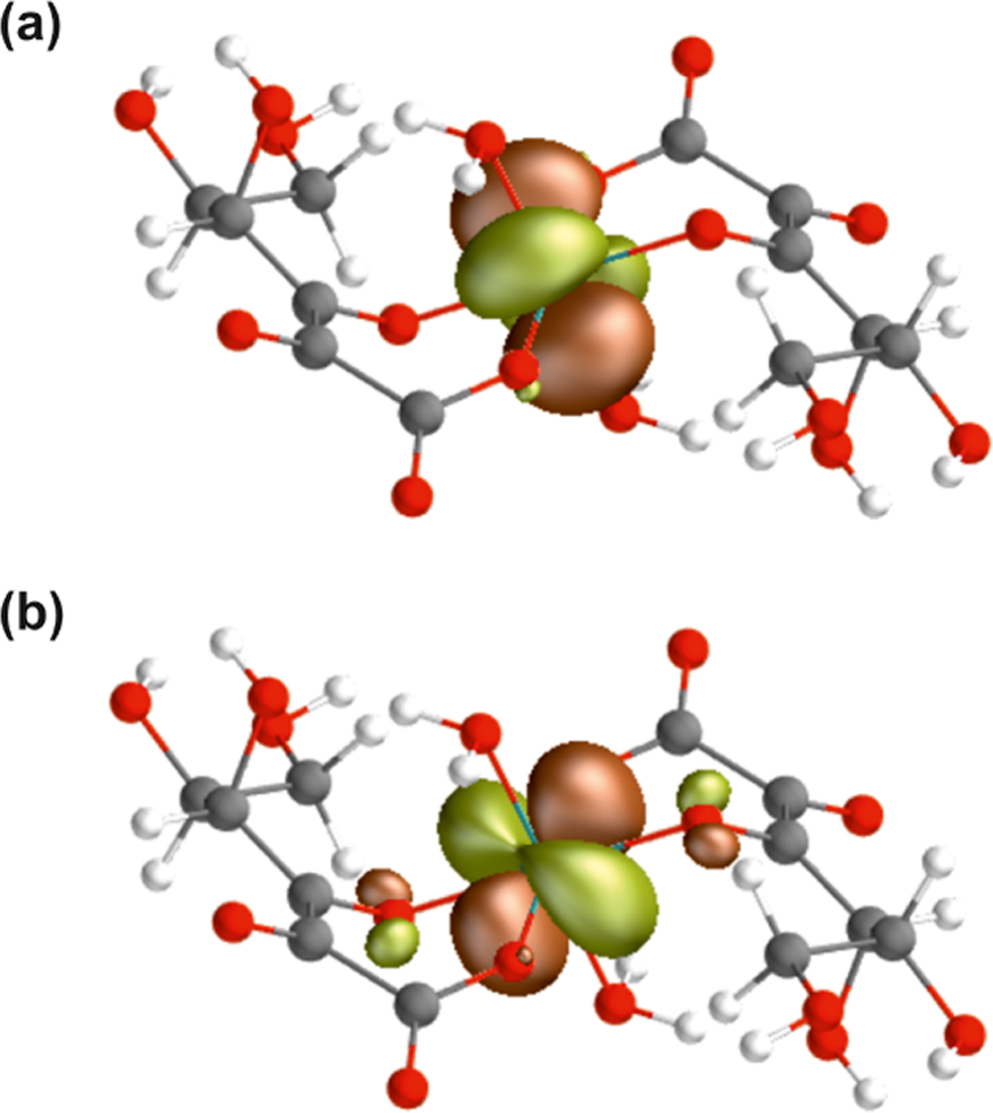
Occupied 4d valence orbitals of the doublet ground state of the [Tc(IV)(Glu_–2H_)_2_(H_2_O)_2_]^2–^ model: (a) doubly occupied 4d_π_ orbital and (b) singly occupied 4d_δ_ orbital.

This changes in the Tc(IV)-gluconate species as studied with the [Tc(IV)(Glu_–2H_)_2_(H_2_O)_2_]^2–^(+4H_2_O) model. From Table 2, it can be clearly seen that the octahedral environment is distorted with the three Tc–O distances varying from 198, 207 to 212 pm and the angles (O–Tc–O) with a maximum deviation of 7° from 90°. This has severe consequences for the ground state. One of the three low-lying 4d orbitals gets doubly occupied, and the total spin is reduced to *S* = 1/2 (see Figure 5a). Hence, the ground state of the [Tc(IV)(Glu_–2H_)_2_(H_2_O)_2_]^2–^(+4H_2_O) model is not a quartet, but a doublet state with a (4d_π_)^2^(4d_δ_)^1^ occupation.^a^ The occupied 4d orbitals are shown in Figure 6. This is the reason why the Tc L_3_-edge XANES spectra differ so strongly between the Tc(IV)O_2_(am,hyd) hydrous oxide reference sample and sample B^7^ with the Tc(IV)-gluconate species, and it has severe consequences for the core-excited states as well (see Section 3.6.2). Following the electronic excitation 2p_3/2_ → 4d, the electronic structure of the core-excited states differs significantly for the [Tc(IV)(Glu_–2H_)_2_(H_2_O)_2_]^2–^(+4H_2_O) model (see Figure 5b) and they are doublet states.

Bauters et al.^24^ reported crystal-field multiplet theory calculations^57,61^ of Tc complexes. Their calculations aim only for the accurate description of the core-excited states with a semiempirical approach and are not suited for an accurate description of the ground state. Here we show that for Tc systems, accurate relativistic multireference all-electron *ab initio* calculations providing a faithful characterization of the ground state are required to obtain the correct understanding of the observed Tc L_3_-edge XANES spectra. The reason for these originates from the complicated electronic structure of Tc(IV) complexes due to the 4d^3^ valence shell occupation with an a priori unknown ground state, which depends strongly on the coordination environment of Tc(IV).

Multireference calculations allow for a thorough investigation of all of the possible different configurations arising from the population of the 4d orbitals with three electrons. There are 40 doublet and 10 quartet low-lying states in A_g_ symmetry. The large number of possible states requires the consideration of all states for both spin couplings (*S* = 1/2 and 3/2) together in two separate calculations, which is possible with neither DFT (with the exception of ROCIS-DFT that is specially adapted for transition metals)-based methods nor the previously mentioned semiempirical approaches.

#### 3.6.2 Core-Excited States

The Tc L_3_-edge XANES spectra of Tc(IV)O_2_(am,hyd) display one main peak (see Figure 5 in ref (7)). But as the analysis of the XANES spectra by Dardenne et al.^7^ shows, it results from the superposition of transitions to two different groups of core-excited states (see Figure 9 in ref (7)).

**Figure 7:**
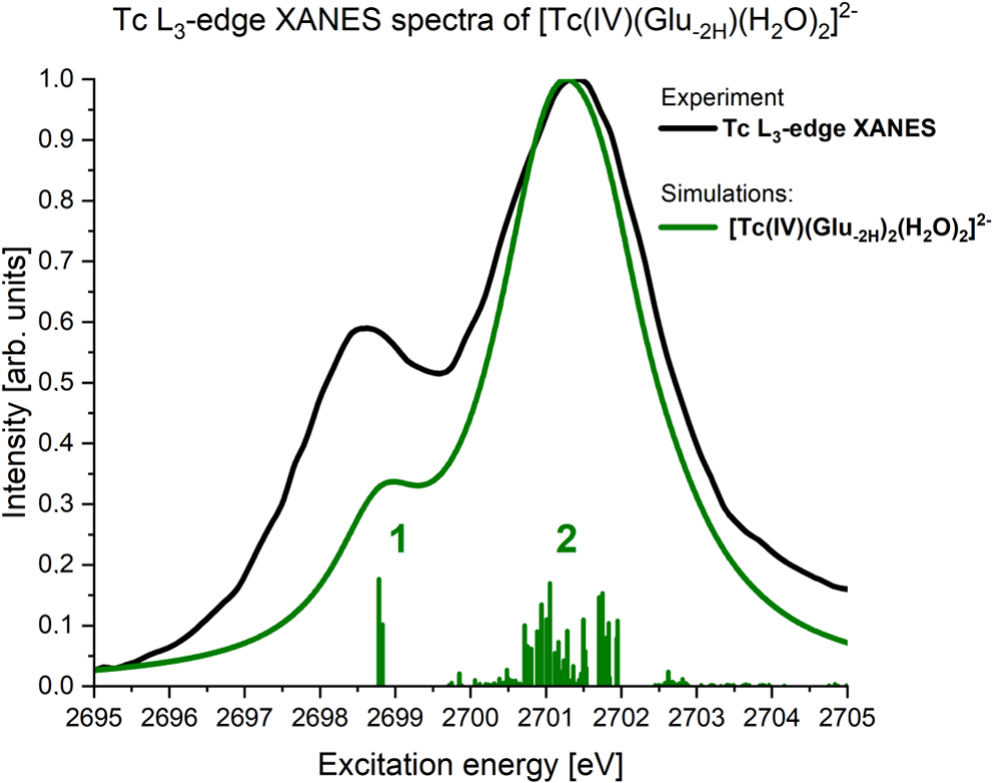
Simulated Tc L_3_-edge XANES spectra of [Tc(IV)(Glu_–2H_)_2_(H_2_O)_2_]^2–^ together with the most intense contributions of different core excitations from groups 1 and 2 shown as vertical bars, giving the excitation energy and intensity of the core excitations.

The simulated Tc L_3_-edge XANES spectra of the Tc(IV)-gluconate species are shown in Figure 7 together with the energies and the oscillator strengths of the most intense transitions. It clearly features a double peak structure, labeled 1 and 2, and it is the superposition of transitions to two groups of core-excited states (see Figures 5b and 7).

The core-excited states belonging to group 1 have a lower energy compared to group 2 and the core-excited states of group 1 are characterized by a (4d_π_)^2^(4d_δ_)^2^ valence orbital occupation. The (4d_δ_) orbital that is occupied with a single electron in the ground state gets doubly occupied (see Figure 5b) in the core-excited states belonging to group 1.

The occupation patterns of the core-excited states of group 2 are quite different (Figure 5b). The other 4d orbitals that remained unoccupied in the doublet ground state are occupied in this group as well. The excitations from the ground state into this group of core-excited states give rise to the more pronounced second peak in Figure 7. In general, the core-excited states of both groups have very strong multireference character.

This is similar to the manifold of core-excited states of the Tc(IV)O_2_(am,hyd) hydrous oxide as reported in ref (7). But the number of transitions, the oscillator strengths, and the energetic separation of the two groups for Tc(IV)O_2_(am,hyd) are different and therefore give rise to only one intense peak.

It is very interesting that the Tc K-edge XANES spectra of sample B containing Tc(IV)-gluconate species and the hydrous oxide Tc(IV)O_2_(am,hyd) reference sample differ only slightly whereas the two corresponding Tc L_3_-edge XANES spectra reveal completely different patterns (see Figures 4 and 5 in ref (7)). The Tc L_3_-edge white line position depends directly on the number of 4d electrons that are probed directly by Tc L_3_-edge XANES. Since the ground-state occupation in both states is different, the Tc L_3_-edge XANES of Tc(IV)-gluconate and Tc(IV)O_2_(am,hyd) hydrous oxide reference sample differ considerably. Although Tc K-edge XANES probes in general the mixing of 5p and 4d orbitals and therefore only indirectly the 4d shell, this is not the case for the Tc(IV)-gluconate and Tc(IV)O_2_(am,hyd) hydrous oxide because of the inversion symmetry *C*_*i*_ in both systems. Therefore, Tc K-edge XANES is less sensitive to different ground-state configurations probed at the Tc L_3_-edge for the two species.

## 4 Conclusions

We characterized the previously unknown Tc(IV)-gluconate species observed in ref (7) in a three-step procedure: (1) we developed chemical models of the previously unknown Tc(IV)-gluconate species based on solubility experiments combined with X-ray spectroscopy to justify the considered stoichiometry.^7,16^ (2) The equilibrium structures of all of the proposed chemical models were optimized with DFT and/or MP2 and compared to experimental EXAFS results. (3) All of the structures with an excellent agreement with the measured values were further selected for subsequent calculations and simulations of the Tc L_3_-edge XANES spectra of these models with the RASPT2 method.

We compared the averaged Tc–O distances of the equilibrium structures with the experimental EXAFS results of Lukens et al.^63^ and Dardenne et al.^7^ and the simulated Tc L_3_-edge XANES spectra with the corresponding data reported by Dardenne et al.^7^ This three-step procedure singled out one chemical model as the most likely candidate for the Tc(IV)-gluconate species in sample B:^7^ [Tc(IV)(Glu_–2H_)_2_(H_2_O)_2_]^2–^(+4H_2_O) (without and with additional water added), in agreement with the interpretation of Lukens et al.^63^ This highlights the success of our completely independent *ab initio* approach for the characterization of the unknown Tc(IV)-gluconate species, which arrives at the same result as Lukens et al.^63^

Similarly, we identified the [Tc(V)O(Glu_–H_)_2_]^–^ structure as the most likely candidate for the Tc(V)-gluconate species in sample A of ref (7).

We note the different protonation degrees of gluconate in the Tc(IV) and Tc(V) complexes investigated in this work, i.e., [Tc(IV)(Glu_–2H_)_2_]^−2^ and [Tc(V)O(Glu_–H_)_2_]^−^. It appears a priori counterintuitive that the Tc(V) cation is less acidic than Tc(IV), thus promoting a lower degree of deprotonation of the alcohol groups of gluconate. This is expected due to the participation of the Tc(V)O^3+^ and Tc^4+^ moieties in the complexation reaction, as confirmed by EXAFS evaluation of the Tc K-edge data.

The calculations and simulations of the Tc L_3_-edge XANES spectra are confirmed as a very sensitive tool for speciation of the Tc(IV) complexes. Furthermore, we obtained detailed information about the electronic structure of the ground as well as the core-excited states and could clearly explain why the Tc L_3_-edge XANES spectra of the sample containing the Tc(IV)-gluconate species and the Tc(IV)O_2_(am,hyd) hydrous oxide reference sample differ so strongly. The [Tc(IV)(Glu_–2H_)_2_(H_2_O)_2_]^2–^(+4H_2_O) ground state of the Tc(IV)-gluconate species is a doublet state whereas the ground state of Tc(IV)O_2_(am,hyd) in the reference sample is a quartet state.^7^

This is a very important result, since XANES is sensitive to the oxidation state and routinely used as a fingerprinting technique to analyze the oxidation state of Tc. As shown here, the L_3_-edge XANES spectra for the same oxidation state can differ significantly due to the change of the ground state caused by the different chemical environment of the Tc(IV) ion. It must be emphasized that this study requires a suitable multireference method to guarantee a proper and accurate description of the ground state. Methods that are not capable doing this, like some DFT-based methods^33,36,43^ or semiempirical methods^53−62^ applied to X-ray spectroscopy, cannot provide this capability to identify the exact electronic structure of the ground state that was the crucial information to understand the difference between the Tc L_3_-edge XANES spectra of the sample with Tc(IV)-gluconate species and the Tc(IV)O_2_(am,hyd) hydrous oxide reference sample.

## Supporting Information Available

Equilibrium structures of Tc(IV)-gluconate structures 1–4 with charge *q* = −1, important additional experimental solubility data for the speciation of the Tc(IV)-gluconate species, and technical details of the calculations (PDF)

## Footnotes

a The *z*-axis in Figure 1 is oriented along the line O–Tc–O with the oxygens belonging to the water molecules interacting with Tc. In a good approximation, the doubly occupied orbital corresponds to a 4d_π_ and the singly occupied orbital to a 4d_δ_ if rotational symmetry with respect to the *z*-axis (*D*_∞*h*_ point group symmetry) is assumed.
