# Supplementary material for: Ab Initio Speciation of Tc-Gluconate Complexes in Aqueous Systems
Source: Inorg Chem. 2025 Mar 9;64(11):5412–23. doi: 10.1021/acs.inorgchem.4c05115 (PMC11938344; doi:10.1021/acs.inorgchem.4c05115)
Supplement: Supplementary file 1 — ic4c05115_si_001.pdf [file ic4c05115_si_001.pdf]

Supporting information
Ab initio speciation of Tc-gluconate complexes in aqueous systems

Robert Polly,* Kathy Dardenne, Sarah Duckworth, Xavier Gaona, Tim Pruessmann, Jörg Rothe, Marcus Altmaier, and Horst Geckeis

Karlsruher Institut für Technologie (KIT), Campus Nord, Institut für Nukleare Entsorgung (INE), Hermann von Helmholtzplatz 1, 76344 Eggenstein-Leopoldshafen, Germany

E-mail: polly@kit.edu

February 20, 2025

S1 Optimized structures of Tc(IV)-gluconate models 1-4 (see Table 1) with $q = -1$

Table S1: Optimized structures of Tc(IV)-gluconate models 1-4 (see Table 1) with $q = -1$. The results of DFT(BP86) and MP2 calculations are shown (Bond lengths in pm).

Theoretical results							
Structure	label	method	[Tc=O]	Tc-O(COO^-)	Tc-O(CO^-)	(OH_2/OH^-)	average
$[Tc(IV)(Glu_{-2H})_1]^{1-}$	1	DFT	170	194	195	–	186
$[Tc(IV)(Glu_{-2H})_1]^{1-}+5H_2O$	2	DFT	173	197	196	226	198
$[Tc(IV)(OH)(Glu_{-H})_1]^{1-}O$	3	DFT	173	206	201	195	194
$[Tc(IV)(OH)_2(Glu)_1]^{1-}O$	4	DFT	174	205	193	–	194
Experimental results							
							average
Sample B (see Ref.[1])							201[1,2]

S2 Tc(IV) solubility data in the presence of gluconate

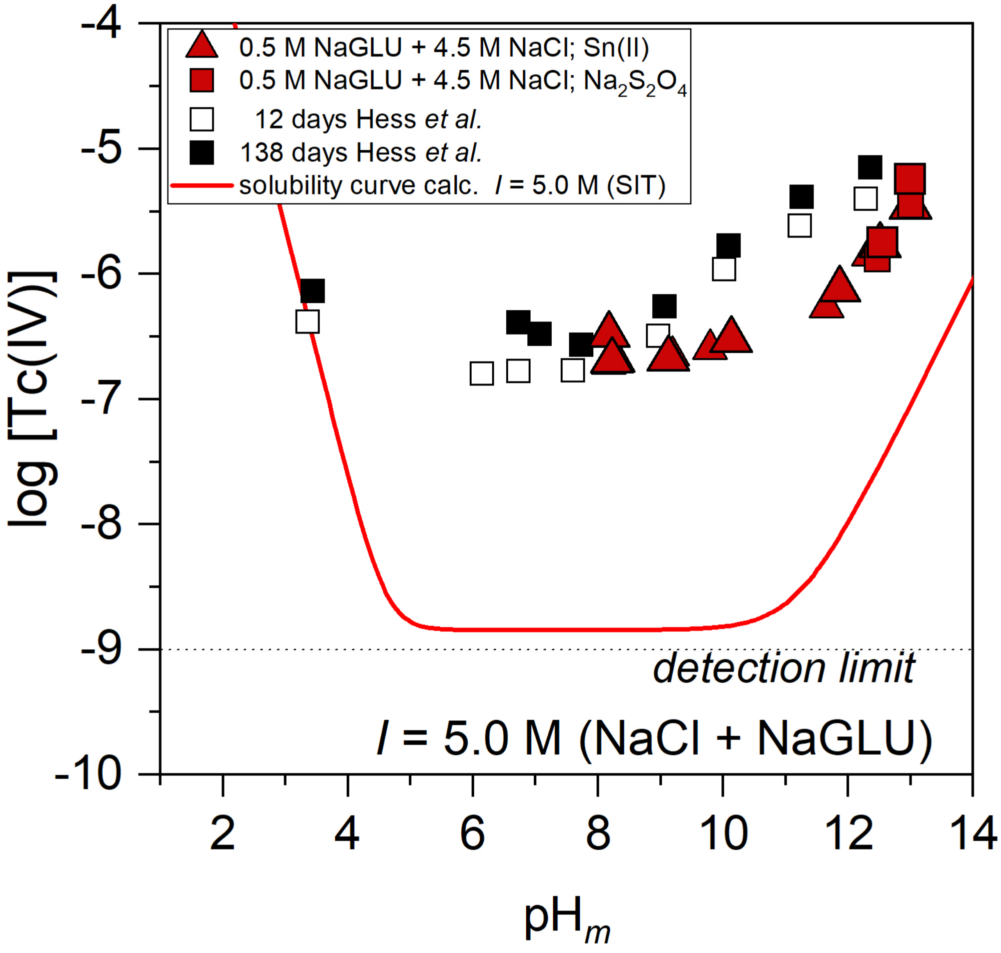

Figure S1: Solubility data of TcO_2(am, hyd) as a function of pH at various gluconate concentrations. Experimental data as reported in Hess et al.[3] and Duckworth.[4] Solid lines correspond to thermodynamically calculated Tc(IV) solubility at I = 0.1 M and 5.0 M NaCl in the absence of Gluconate, using the current NEA-TDB selection.

S3 Calculations and simulations of Tc L_3-edge XANES spectra

We used the restricted active space (RASSCF) method[5] and for the inclusion of the dynamical correlation second-order perturbation theory RASPT2[6] with an IPEA shift of 0.0. The core-excited states of the Tc L_3-edge manifold correspond to $2p_{3/2} \rightarrow 4d$ core-excitations. Both the 2p core- (RAS1) as well 4d valence (RAS2/3) orbitals are in the active space. In total, we had 9 active electrons distributed in the active space. We allowed one hole in the RAS1 space and a maximum of four electrons in the RAS3 space. Both scalar relativistic and spin-orbit coupling were accounted for. Spin-orbit interactions were calculated in the restricted-active-space state-interaction (RASSI) scheme.[6] For the spin-orbit interaction calculations, all relevant spin states were included in the calculations. As basis sets, we used the ANO-VDZ basis sets[7,8] available in MOLCAS. We performed the calculations using the C_i symmetry of the Tc(IV)-gluconate complexes.

S4 Calculation of the Tc L_3-edge XANES spectra of the selected Tc(IV)-gluconate models

Table S2: Spin free states included in the calculations of the Tc L_3-edge X-ray absorption near-edge spectroscopy (XANES) spectra of the various chemical models

	holes 2p shell	electrons 4d valence	total spin S	number of spin-free states
Lowest doublet state	0	3	1/2	1
Lowest quartet state	0	3	3/2	1
core excited doublet states	1	4	1/2	285
core excited quartet states	1	4	3/2	150
core excited sextet states	1	4	5/2	15

In total, there are $285 + 150 + 15 = 450$ spin free states included in the calculation. They correspond to $2 \cdot 285 + 4 \cdot 150 + 6 \cdot 15 = 1260$ spin-orbit coupled states (840 belonging to the Tc L_3-edge manifold and 420 belonging to the Tc L_2-edge manifold).

S5 References

References

(1) Dardenne, K.; Duckworth, S.; Gaona, X.; R.Polly,; Schimmelpfennig, B.; Pruessmann, T.; Rothe, J.; Altmaier, M.; Geckeis, H. A Combined Study of Tc Redox Speciation in Complex Aqueous Systems: Wet-Chemistry, Tc K-/L_3-Edge X-ray Absorption Fine Structure, an Ab Initio Calculations. *Inorganic Chemistry* **2021**,

(2) Lukens, W. W.; Shuh, D. K.; Schroeder, N. C.; Ashley, K. R. Identification of the Non-Pertechnetate Species in Hanford Waste Tanks, Tc(I)-Carbonyl Complexes. *Environ. Sci. Technol.* **2004**, *38*, 229.

(3) Hess, N. J.; Xia, Y.; Felmy, A. R. *in Nucl. Waste Manag., American Chemical Society* **2006**, *286*.

(4) Duckworth, S. Aquatische Chemie von Technetium in Anwesenheit anorganischer und organischer Liganden unter endlagerrelevanten Bedingungen. Ph.D. thesis, Karlsruhe Institute of Technology, Institute of nuclear waste disposal (INE), 2021.

(5) Malmqvist, P.-Å.; Rendell, A.; Roos, B. O. The restricted active space self-consistent-field method, implemented with a split graph unitary-group approach. *J. Phys. Chem.* **1990**, *94*, 5477.

(6) Malmqvist, P.-Å.; Pierloot, K.; Shahi, A. R. M.; Cramer, C. J.; Gagliardi, L. The Restricted Active Space Followed by Second-Order Perturbation Theory Method:

Theory and Application to the Study of CuO2 and Cu2O2 Systems. *J. Chem. Phys.* **2008**, *128*, 204109.

(7) Roos, B. O.; Lindh, R.; Malmqvist, P.-Å.; Veryazov, V.; Widmark, P.-O. Molecular dynamics simulations of the interactions between water and inorganic solids. *J. Phys. Chem. A* **2004**, *108*, 2851.

(8) Roos, B. O.; Lindh, R.; Malmqvist, P.-Å.; Veryazov, V.; Widmark, P.-O. New relativistic ANO basis sets for transition metal atoms. *J. Phys. Chem. A* **2005**, *109*, 6575.
